# Supplementary material for: Prognostic Significance of GPR55 mRNA Expression in Colon Cancer
Source: Int J Mol Sci. 2022 Apr 20;23(9):4556. doi: 10.3390/ijms23094556 (PMC9106053; doi:10.3390/ijms23094556)
Supplement: Supplementary file 1 [file ijms-23-04556-s001.zip › Supplementary Table S1.pdf]

Supplementary Table S1. Comparative analysis of average survival time after surgery in CC patients with GPR55(-) and GPR55(+) without prognostic value

Patient Group	Category	5 Years Follow-Up after Surgery			12 Years Follow-Up after Surgery		
		Disease-free Survival [a]			Disease-free Survival [a]		
		Average (Months)	Difference (Months)	*P*-value	Average (Months)	Difference (Months)	*P*-value
CEA[c]	*GPR55* (–) [b]	35	7	0.586	56	8	0.779
	GPR55(+)	42			64		
CXCL16 [d]	*GPR55* (–)	56	2	0.751	112	3	0.621
	GPR55(+)	54			109		
CXCL17 [e]	*GPR55* (–)	55	1	0.949	109	7	0.169
	GPR55(+)	56			116		
GPR35 V2/3 [f]	*GPR55* (–)	52	2	0.649	101	9	0.167
	GPR55(+)	54			110		
LGR5 [g]	*GPR55* (–)	56	1	0.855	109	3	0.328
	GPR55(+)	55			112		

(a) Mean survival time after surgery calculated by cumulative survival analysis according to Kaplan-Meier model
(b) CC patients divided into two groups, GPR55(–) and GPR55(+) using a cutoff of 0.1365 mRNA copies/ 18S rRNA unit
(c) CC patient group with CEA mRNA levels above 3.67 mRNA copies/18S rRNA unit
(d) CC patient group with CXCL16 mRNA levels below 7.2 mRNA copies/18S rRNA unit
(e) CC patient group with CXCL17 mRNA levels above 0.0003 mRNA copies/18S rRNA unit
(f) CC patient group with GPR35 V2/3 mRNA levels below 11.7 mRNA copies/18S rRNA unit
(g) CC patient group with LGR5 mRNA levels below 0.062 mRNA copies/18S rRNA unit
