# Supplementary figures and images for: Prognostic Significance of GPR55 mRNA Expression in Colon Cancer
Source: Int J Mol Sci. 2022 Apr 20;23(9):4556. doi: 10.3390/ijms23094556 (PMC9106053; doi:10.3390/ijms23094556)

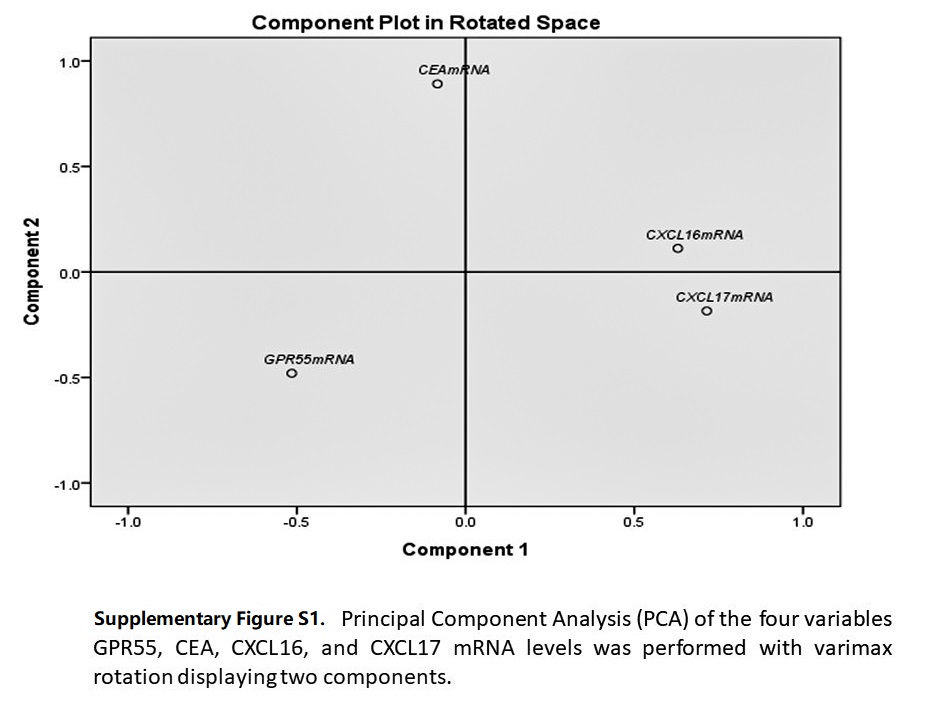

Supplement: Supplementary file 1 [file ijms-23-04556-s001.zip › Supplementry figure S1.jpg]
